# Supplementary material for: Assessing the ecological validity of soundscape reproduction in different laboratory settings
Source: PLoS One. 2022 Jun 27;17(6):e0270401. doi: 10.1371/journal.pone.0270401 (PMC9236251; doi:10.1371/journal.pone.0270401)
Supplement: S1 Table — (DOCX) [file pone.0270401.s001.docx]

|  | Test statistic | p-value |
| --- | --- | --- |
| Day | 6.782 | 0.543 |
| Time | 5.142 | 0.725 |
| Day x Time | 5.389 | 0.692 |
| Location | 6.556 | 0.556 |
| Day x Location | 6.361 | 0.583 |
| Times x Location | 9.692 | 0.339 |
| Day x Time x Location | 12.002 | 0.208 |
