# Supplementary material for: Assessing the ecological validity of soundscape reproduction in different laboratory settings
Source: PLoS One. 2022 Jun 27;17(6):e0270401. doi: 10.1371/journal.pone.0270401 (PMC9236251; doi:10.1371/journal.pone.0270401)
Supplement: S2 Table — (DOCX) [file pone.0270401.s002.docx]

|  | Pleasant | Appropriate | Monotonous | Vibrant | Chaotic | Calm | Eventful | Restorative |
| --- | --- | --- | --- | --- | --- | --- | --- | --- |
| Day | 1.982 | 0.031 | 2.335 | 0.208 | 0.737 | 1.143 | 0.001 | 0.345 |
| Time | 0.108 | 0.221 | 0.66 | 0.042 | 0.468 | 0.364 | 0.663 | 2.616 |
| Day x Time | 0.847 | 0.243 | 0.117 | 0.325 | 0.577 | 2.061 | 1.158 | 0.061 |
| Location | 0.124 | 0.009 | 3.038 | 0.07 | 0.055 | 0.174 | 0.359 | 2.728 |
| Day x Location | 0.595 | 0.673 | 0.035 | 0.827 | 0.626 | 0.534 | 0.098 | 2.973 |
| Time x Location | 0.428 | 0.216 | 4.83 | 1.787 | 0.824 | 0.000 | 0.318 | 1.289 |
| Day x Time x Location | 1.123 | 0.081 | 2.562 | 0.186 | 4.421 | 2.127 | 0.409 | 1.092 |
