# Supplementary material for: Assessing the ecological validity of soundscape reproduction in different laboratory settings
Source: PLoS One. 2022 Jun 27;17(6):e0270401. doi: 10.1371/journal.pone.0270401 (PMC9236251; doi:10.1371/journal.pone.0270401)
Supplement: Attachment — Submitted filename: Comments for PONE-21-18471.pdf [file pone.0270401.s003.pdf]

Ref. Review: PONE-D-21-18471

Paper Title: Assessing the ecological validity of soundscape reproduction in different laboratory Settings

Dear Authors and Editor,

Based on the text exposed in the entitled paper: "Assessing the ecological validity of soundscape reproduction in different laboratory Settings", I recommend major revisions before acceptance and publishing on the Plos One Journal.

The work presented in this paper proposed exploring the ecological validity of soundscape reproduction in the laboratory using first order Ambisonics and different modes of questionnaire administration.

The suggested work has the potential to be an excellent example of a listening test using first-order Ambisonics equipment for soundscape studies.

For the improvement of the manuscript quality, the following suggestions should be observed:

## Methods:

### Procedure:

1. There was no training period. How to be sure if the participants understood all asked tasks in each experiment procedure?

2. Regarding the time control in the computer-based experiment, the authors informed that the time was not controlled on the same basis in the paper-and-pen experiment. For sure, the time control adds some stress to the listeners and could be a bias in this experiment. The optimal situation should be adding time control for the paper-and-pen experiment or removing the control of time for the computer-based experiment. Could you please check the mentioned bias and ensure that the related research question will show reliable results?

### Statistical analysis:

3. Could the author show a flowchart with all statistical procedures, indicating how the statistical analysis was used for each research question and expected output?

## Results:

4. What about the CFA assumptions? Could you please inform in the text if all assumptions to conduct a CFA were followed?

   https://www.statisticssolutions.com/free-resources/directory-of-statistical-analyses/confirmatory-factor-analysis/

5. Could you please also show if the MANOVA assumptions were fulfilled?
