# Supplementary material for: Assessing the ecological validity of soundscape reproduction in different laboratory settings
Source: PLoS One. 2022 Jun 27;17(6):e0270401. doi: 10.1371/journal.pone.0270401 (PMC9236251; doi:10.1371/journal.pone.0270401)
Supplement: Attachment — Submitted filename: Comments for PONE-21-18471R1.pdf [file pone.0270401.s005.pdf]

Ref. Review: PONE-D-21-18471R1

Paper Title: Assessing the ecological validity of soundscape reproduction in different laboratory Settings

Dear Authors and Editor,

Based on the text exposed in the entitled paper: "Assessing the ecological validity of soundscape reproduction in different laboratory Settings", I recommend **minor revisions** before acceptance and publishing in the Plos One Journal.
The work presented in this paper proposed exploring the ecological validity of soundscape reproduction in the laboratory using first-order Ambisonics and different modes of questionnaire administration.

The suggested work has the potential to be an excellent example of a listening test using first-order Ambisonics equipment for soundscape studies.

For the improvement of the manuscript quality, the following suggestions should be observed:

Results:

Could you please also show if the MANOVA assumptions were fulfilled?
Authors' answer: The semi-parametric repeated-measures MANOVA we used does not rely on assumptions (see Friedrich et al., 2017, i.e., ref. [68]).

Could you please complement the text with the argumentation of the ref [68]? For example, I suggest: "preserving their general applicability for all kinds of data in factorial repeated measures and split-plot designs" [68], eliminating the MANOVA assumptions fulfilment.
