# Supplementary material for: Assessing the ecological validity of soundscape reproduction in different laboratory settings
Source: PLoS One. 2022 Jun 27;17(6):e0270401. doi: 10.1371/journal.pone.0270401 (PMC9236251; doi:10.1371/journal.pone.0270401)
Supplement: Attachment — Submitted filename: response to reviewers.docx [file pone.0270401.s006.docx]

The authors thank the reviewers for their comments. We trust that we adequately addressed the last remaining concern mentioned (see tracked changes in the manuscript). We also reviewed all references.

Reviewer #1: Excellent paper, important results, rigorous science. A pleasure to read this. Congratulations. Looking forward to seeing where you go next with this.

Reviewer #2: The authors have answered all my questions and I think this manuscript suitable for publication now.

Reviewer #3: Ref. Review: PONE-D-21-18471R1
Paper Title: Assessing the ecological validity of soundscape reproduction in different laboratory Settings

Dear Authors and Editor,
Based on the text exposed in the entitled paper: "Assessing the ecological validity of soundscape reproduction in different laboratory Settings", I recommend minor revisions before acceptance and publishing in the Plos One Journal.
The work presented in this paper proposed exploring the ecological validity of soundscape reproduction in the laboratory using first-order Ambisonics and different modes of questionnaire administration.
The suggested work has the potential to be an excellent example of a listening test using first-order Ambisonics equipment for soundscape studies.
For the improvement of the manuscript quality, the following suggestions should be observed:

Results:

Could you please also show if the MANOVA assumptions were fulfilled?
Authors' answer: The semi-parametric repeated-measures MANOVA we used does not rely on assumptions (see Friedrich et al., 2017, i.e., ref. [68]).

Could you please complement the text with the argumentation of the ref [68]? For example, I suggest:
"preserving their general applicability for all kinds of data in factorial repeated measures and split-plot designs" [68], eliminating the MANOVA assumptions fulfilment.

Changed as requested – see lines 445-447
